# Supplementary material for: Role of the MDR Efflux Pump AcrAB in Epithelial Cell Invasion by Shigella flexneri
Source: Biomolecules. 2023 May 11;13(5):823. doi: 10.3390/biom13050823 (PMC10216353; doi:10.3390/biom13050823)
Supplement: Supplementary file 1 [file biomolecules-13-00823-s001.zip › Table S1.pdf]

Table S1. Oligonucleotides used in this study.

Chromosomal deletions	
Name	5′-3′ sequence
SfAF	ACTTTTGACCATTGACCAATTTGAAATCGGACACTCGAGGTTTACATATGTGTAGGCTGGAGCTGCTTCG
SfBR	AAAGAATACCGGAACGAAGAAGATTGCCAGTACCGTTGCGATATGAATATCCTCCTTAGT
SfAR	TAGGCATGTCTTAACGGCTCCTGTTTAAGTTAAGACTTGGACTGTTCAGGCATATGAATATCCTCCTTA
SfBF	CCTGAACAGTCCAAGTCTTAACTTAAACAGGAGCCGTTAAGACATGCCTATGTAGGCTGGAGCTGCTTCG
Plasmids construction	
Name	5′-3′ sequence
pSfacrBF	NNNGGATCCATGCCTAATTTCTTTATCGATCGC
pSfacrBR	NNNGGATCCGATCCTGAGTTGGTGGTTCAATTACT
pSfacrB$_{D408A}$ F	CATCGGCTTGTTGGTGGATGCCGCTATCGTTGTGGTAGAAA
pSfacrB$_{D408A}$ R	TTTCTACCACAACGATAGCGGCATCCACCAACAAGCCGATGG
