# Supplementary figures and images for: Role of the MDR Efflux Pump AcrAB in Epithelial Cell Invasion by Shigella flexneri
Source: Biomolecules. 2023 May 11;13(5):823. doi: 10.3390/biom13050823 (PMC10216353; doi:10.3390/biom13050823)

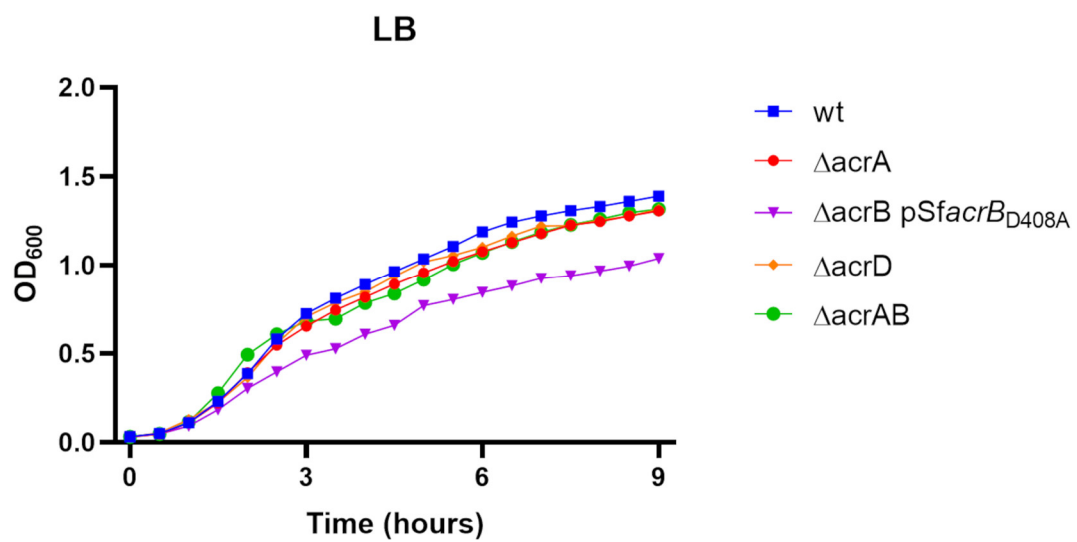

**Figure S1.** Growth curves of M90T wt and derivative strains.

Supplement: Supplementary file 1 [file biomolecules-13-00823-s001.zip › Figure S1.pdf]
